# Optimizing Clinical Trial Eligibility Design Using Natural Language Processing Models and Real-World Data: Algorithm Development and Validation

**DOI:** 10.2196/50800

**Published:** 2024-07-29

**Authors:** Kyeryoung Lee, Zongzhi Liu, Yun Mai, Tomi Jun, Meng Ma, Tongyu Wang, Lei Ai, Ediz Calay, William Oh, Gustavo Stolovitzky, Eric Schadt, Xiaoyan Wang

**Affiliations:** 1 GendDx (Sema4) Stamford, CT United States; 2 Icahn School of Medicine at Mount Sinai New York, NY United States

**Keywords:** natural language processing, real-world data, clinical trial eligibility criteria, eligibility criteria–specific ontology, clinical trial protocol optimization, data-driven approach

## Abstract

**Background:**

Clinical trials are vital for developing new therapies but can also delay drug development. Efficient trial data management, optimized trial protocol, and accurate patient identification are critical for reducing trial timelines. Natural language processing (NLP) has the potential to achieve these objectives.

**Objective:**

This study aims to assess the feasibility of using data-driven approaches to optimize clinical trial protocol design and identify eligible patients. This involves creating a comprehensive eligibility criteria knowledge base integrated within electronic health records using deep learning–based NLP techniques.

**Methods:**

We obtained data of 3281 industry-sponsored phase 2 or 3 interventional clinical trials recruiting patients with non–small cell lung cancer, prostate cancer, breast cancer, multiple myeloma, ulcerative colitis, and Crohn disease from ClinicalTrials.gov, spanning the period between 2013 and 2020. A customized bidirectional long short-term memory– and conditional random field–based NLP pipeline was used to extract all eligibility criteria attributes and convert hypernym concepts into computable hyponyms along with their corresponding values. To illustrate the simulation of clinical trial design for optimization purposes, we selected a subset of patients with non–small cell lung cancer (n=2775), curated from the Mount Sinai Health System, as a pilot study.

**Results:**

We manually annotated the clinical trial eligibility corpus (485/3281, 14.78% trials) and constructed an eligibility criteria–specific ontology. Our customized NLP pipeline, developed based on the eligibility criteria–specific ontology that we created through manual annotation, achieved high precision (0.91, range 0.67-1.00) and recall (0.79, range 0.50-1) scores, as well as a high *F*_1_-score (0.83, range 0.67-1), enabling the efficient extraction of granular criteria entities and relevant attributes from 3281 clinical trials. A standardized eligibility criteria knowledge base, compatible with electronic health records, was developed by transforming hypernym concepts into machine-interpretable hyponyms along with their corresponding values. In addition, an interface prototype demonstrated the practicality of leveraging real-world data for optimizing clinical trial protocols and identifying eligible patients.

**Conclusions:**

Our customized NLP pipeline successfully generated a standardized eligibility criteria knowledge base by transforming hypernym criteria into machine-readable hyponyms along with their corresponding values. A prototype interface integrating real-world patient information allows us to assess the impact of each eligibility criterion on the number of patients eligible for the trial. Leveraging NLP and real-world data in a data-driven approach holds promise for streamlining the overall clinical trial process, optimizing processes, and improving efficiency in patient identification.

## Introduction

### Background

Clinical trials are crucial for developing new therapies, but they require significant resources and can introduce delays in drug development, leading to increased costs [1,2]. Complex and restrictive eligibility criteria hinder patient enrollment, impacting target goals, timelines, and ultimately patient well-being [3-5]. This issue is particularly notable in cancer trials with poor recruitment and high failure rates [6-8] because >80% of the trials fail to meet their initial target accruals and timelines [6,9]. In addition, overly restrictive eligibility criteria limit the representation of the broader patient population, reducing real-world applicability and treatment impact [10-13]. Nonetheless, the practice of trials reusing complicated eligibility criteria without a clear rationale is a common one [14], despite the minimal impact on trial outcomes [15]. Liu et al [15] demonstrated that broadening eligibility criteria using a data-driven approach can benefit initially excluded patients. A comprehensive and standardized eligibility criteria knowledge base that is compatible with real-world data can address these challenges. Such a knowledge base optimizes trial protocol design, improves patient enrollment, enhances the reliability and applicability of evidence synthesis, and fosters the efficient development of new therapies. Furthermore, it enables opportunities such as generating synthetic control arms (SCAs) for single-arm clinical trials using electronic health records (EHRs) [16-18].

The importance of semantically representing eligibility criteria interoperable with EHRs has been highlighted in multiple studies [19-21]. Converting free-text eligibility criteria to computable formats poses challenges, addressed by a range of natural language processing (NLP) techniques and transformer models [22-26]. An NLP interface, Criteria2Query, enables computable queries for eligible cohort identification using EHRs [27]. This tool supports clinical trial knowledge base development, enhancing EHR interoperability and scalability for efficient eligibility criteria knowledge engineering [28]. Manually annotated data sets such as “Chia, a large annotated corpus of clinical trial eligibility criteria” [29] and the “Leaf Clinical Trials corpus, the largest and most comprehensive human-annotated corpus of publicly available clinical trials eligibility criteria” [30] have significantly enhanced NLP model training and the development of effective query structures. Despite significant progress in bridging the gap between eligibility criteria and EHRs, limitations persist in accurately representing the granularities of eligibility criteria and real-time eligible patient number checks [20,31,32]. Using varying hierarchical levels of medical concepts, whether as hypernyms or hyponyms, presents one of the challenges when aligning eligibility criteria with EHRs; for instance, numerous trial eligibility criteria use hypernyms, which encompass a group of related medical concepts, such as *cardiovascular disease*. Conversely, the patient problem list within the EHR specifies particular medical conditions or diseases (hyponyms), such as *myocardial infarction*. Establishing a standardized eligibility criteria knowledge base by transforming ambiguous hypernym concepts into computable hyponyms can enhance optimizing trial protocol design and identifying eligible patients through seamless integration with EHR data.

### Objectives

In this study, we aim to create a standardized eligibility criteria knowledge base that seamlessly integrates with EHRs. By using deep learning–based NLP techniques, hypernym concepts in eligibility criteria will be converted to their EHR-compatible hyponyms along with their corresponding values. In addition, the prototype user interface will be developed as a pilot study, enabling the data-driven optimization of clinical trial protocols and the identification of eligible patients through the integration of the eligibility criteria knowledge base and EHRs.

## Methods

### Data Set

We obtained the data from ClinicalTrials.gov, specifically industry-sponsored phase 2 or 3 interventional clinical trials initiated between January 2013 and May 2020. A total of 3281 trials were identified: 817 (24.9%) for non–small cell lung cancer (NSCLC), 649 (19.78%) for prostate cancer (PCa), 1057 (32.22%) for breast cancer (BCa), 447 (13.62%) for multiple myeloma (MM), 160 (4.88%) for ulcerative colitis (UC), and 151 (4.6%) for Crohn disease (CD).

For the development of the prototype interface, we selected a subgroup of patients (n=2775) diagnosed with NSCLC from a previously curated cohort of patients with lung cancer. This cohort was established using the data from Mount Sinai-Sema4 Health System data [33], and patient information was deidentified for the purposes of this study.

### Deep Learning–Based NLP Pipeline Development

Our NLP pipeline consists of 3 modules: ontology construction and manual annotation, model training and pipeline evaluation, and application.

#### Ontology Construction and Manual Annotation

To construct our ontology, we randomly selected 425 eligibility criteria from diverse cancer trials and manually analyzed entities and relations. This manual analysis focused on identifying entities and their relationships. Entities were subsequently categorized into primary and modifier groups, with detailed examples provided in Multimedia Appendices 1 and 2. The primary groups included *demographic*, *diagnosis*, *biomarker*, *disease status*, *prior therapy*, *comorbidity*, *laboratory test*, *vital*, *procedure*, and *other medication*, while the modifier groups included *value*, *condition*, *evidence*, *lines of therapy*, *negation*, *exception*, *grade*, *dose*, and *temporal*. Any entities that did not fall into the primary groups were classified as *other observation*. Furthermore, we defined relations between the entities. The commonly detected relationships between the *primary* and *modifier* groups were (1) *has_value_limit* between *demographic (age) or vital lorlaboratory test* and *value*, (2) *has temporal limit* between *comorbidity or other medication or procedure* and *temporal*, (3) *has_negation* between *observation or biomarker or prior therapy* and *negation,* and (4) *has_exception* between *comorbidities or biomarker or diagnosis* and *exception.* Other relationships included *has_dose limit*, *has_line of therapy limit*, *has_grade_limit*, *has_condition*, and *need_evidence*. The applicability of the ontology was tested on 60 UC and CD trials. Next, we manually annotated 246 eligibility criteria from NSCLC trials and performed model training using Clinical Language Annotation, Modeling, and Processing, which is an NLP toolkit [34].

#### Model Training and Pipeline Evaluation

A multilayer deep learning architecture was implemented for NLP modeling. The first step involved transforming the text into sequential vectors of characterization during the embedding process. These vectors were subsequently input into a bidirectional long short-term memory network, which is an artificial neural network designed for text classification. The bidirectional long short-term memory network was used to recognize patterns in both forward and backward directions [35]. The identified patterns were then passed to the next layer, which used a conditional random field model to compute the prediction probability [36]. The NLP model was trained using annotated criteria, with 80% of the manually annotated gold standard data allocated for training. Model performance was evaluated on a separate validation set (20%) using precision, recall, and *F*_1_-score values:

Precision = TP / (TP + FP) **(1)**

Recall = TP / (TP + FN) **(2)**

*F*_1_-score = 2 × (Precision × Recall) / (Precision + Recall) **(3)**

In equations 1 and 2, TP stands for true positives, FP for false positives, and FN for false negatives.

The manual annotation and training processes were iteratively performed with additional manually annotated notes until the model achieved a *F_1_*-score of >0.8 in the test set (Multimedia Appendix 3). To tailor the pipeline for specific cancer types, a preannotation method using the NSCLC pipeline was implemented for PCa, BCa, and MM for common eligibility criteria such as laboratory test values and comorbidities. Specific eligibility criteria such as biomarkers and treatments were manually annotated for each cancer type: PCa with 124 trials, BCa with 73 trials, and MM with 60 trials.

#### Application

The fully trained named entity recognition and relation models were integrated and applied to annotate the remaining eligibility criteria for the 4 types of cancer studied (BCa, MM, NSCLC, and PCa). The output data included sentences, tokens, parts of speech, entities, negations, and relations.

### Construction of Standardized Eligibility Criteria Knowledge Base Table

The standardized knowledge base was constructed in an *EntityGroup-AttributeName-Value* format, involving 2 key steps: attribute normalization and transforming hypernyms to hyponyms with corresponding values.

#### Attribute Normalization

To normalize attributes, we used a 3-step approach. First, we assigned a Unified Medical Language System concept unique identifier to map synonyms of an entity, such as *estrogen receptor-positive*, *ER-positive*, and *ER+* to the Unified Medical Language System concept unique identifier *C0279754*. Second, we developed a set of rules (Table 1) to map abbreviations (eg, *CrCl* to *creatinine clearance*) and different phrases with the same meaning (eg, ≥*1.5x ULN* [where *ULN* stands for *upper limit of normal*], *greater than or equal to 1.5x ULN*, and ≥*1.5x upper limit of normal*) back to their original text. Finally, 2 domain experts manually curated unnormalized entities.

**Table 1:** Rules for attribute normalization.

Rule and attributes from eligibility criteria			Normalized attributes
**Rules for mapping synonyms**			
	AST^a^, SGOT^b^, aspartate aminotransferase, serum AST	AST	
	ALT^c^, SGPT^d^, alanine aminotransferase, serum ALT	ALT	
	Total bilirubin, serum bilirubin, total bilirubin level, bilirubin level	Total bilirubin	
	Hgb^e^, hemoglobin	Hgb	
	HbA_1c_^f^, hemoglobinA_1c_	HbA_1c_	
	serum creatinine, creatinine, creatinine levels, creatinine level	Serum creatinine	
	ANC^g^, absolute neutrophil count, absolute neutrophil counts, neutrophil count, neutrophil counts, absolute neutrophil	ANC	
	WBC^h^, white blood cells, white blood cell, WBC count, white blood cell count, white blood count, leucocytes	WBC	
	platelets, platelet, platelet count, platelet counts	Platelets	
	CrCl^i^, creatine clearance	CrCl	
	ALP^j^, alkaline phosphatase	ALP	
	ULN^k^, upper limit of normal	ULN	
	LLN^l^, lower limit of normal	LLN	
**Rules related to unit and temporal modifier**			
	less than or equal to, ≤	≤	
	greater than or equal to, ≥	≥	
	greater than, >	>	
	less than, <	<	
	within 4 weeks, within 28 days	within 4 weeks	
	within 2 weeks, within 14 days	within 2 weeks	
	within 3 weeks, within 21 days	within 3 weeks	
	last 6 months, past 6 months, within 6 months, within six months	within 6 months	
	last 3 months, past 3 months, within 3 months, within three months	within 3 months	
	within 2 years, last 2 years, past 2 years	within 2 years	
	within 3 years, last 3 years, past 3 years	within 3 years	
	within 5 years, last 5 years, past 5 years	within 5 years	
	10^9^/L, 10^9^/L, 10^3^/uL, 10^3^/microliter, 1000/uL, 1000/microliter, K/microliter, 10^3^/mm^3^	10^3^/uL	
**Other miscellaneous rules**			
	Case insensitive	—^m^	
	Remove spaces	—	

^a^AST: aspartate aminotransferase.

^b^SGOT: serum glutamic oxaloacetic transaminase.

^c^ALT: alanine transaminase.

^d^SGPT: serum glutamic pyruvic transaminase.

^e^Hgb: hemoglobin.

^f^HbA_1c_: glycated hemoglobin.

^g^ANC: absolute neutrophil count.

^h^WBC: white blood cell.

^i^CrCl: creatinine clearance.

^j^ALP: alkaline phosphatase.

^k^ULN: upper limit of normal.

^l^LLN: lower limit of normal.

^m^Not applicable.

#### Transforming Hypernyms to Hyponyms Along With Corresponding Values

To formalize hypernyms, identified in primary groups such as *laboratory test*, *comorbidity*, biomarker, *prior therapy*, and *other medication*, we used the following approaches: (1) for *adequate organ function* *laboratory test* values, we determined prevalent laboratory test values by analyzing the unique laboratory test values for each test across the trials of the same cancer type that defined the normal organ function; and (2) for *comorbidity*, *biomarker*, *prior therapy*, and *other medication* hypernyms, we collected all example hyponyms described across the trials of the same cancer type.

### Creation of a Prototype Interface for Enhancing Trial Protocol Design Optimization

We developed a prototype interface using the R programming language (R Foundation for Statistical Computing) and the *Shiny* package to enhance trial protocol design optimization. The interface allows users to simulate the number of eligible patients based on specific criteria, including a combination of criteria such as histology, stages, laboratory test values, performance scores, prior line of therapy, and comorbidities. For this pilot study, a subset of patients with NSCLC (n=2775) was selected and deidentified. To ensure consistency and accuracy, we standardized the sample entities found in both the eligibility criteria knowledge base and EHRs using concept codes such as the *International Classification of Diseases*; Logical Observation Identifiers, Names, and Codes (LOINC); and normalized medical prescription codes. In addition, we converted the patients’ absolute laboratory test values to either the upper limit of normal (ULN) or the lower limit of normal based on the provided normal ranges for each specific test. Tables 2 and 3 and Textbox 1 present some examples of normalized concepts and their codes.

**Table 2:** Examples of normalized codes for each concept and normal range of each laboratory test.

Laboratory test	LOINC^a^ code	Normal range
ALT^b^ (SGPT^c^; U/L)	1742-6	7-56
AST^d^ (SGOT^e^; U/L)	1920-8	10-40
Total bilirubin in serum (mg/dL)	1975-2	0.1-1.2
Direct (conjugated) bilirubin in serum (mg/dL)	1968-7	<0.3
Serum creatinine (mg/dL)	2160-0	0.6-1.2 (male), 0.5-1.1 (female)
CrCl^f^ (mL/min)	2164-2	97-137 (male), 88-128 (female)
ANC^g^ (cells/µL)	26499-4	>90 mL/min/1.73 m^2^
Platelets (cells/µL)	777-3	150,000-450,000
Hemoglobin (g/dL)	718-7	12-18

^a^LOINC: Logical Observation Identifiers, Names, and Codes.

^b^ALT: alanine transaminase.

^c^SGPT: serum glutamic pyruvic transaminase.

^d^AST: aspartate aminotransferase.

^e^SGOT: serum glutamic oxaloacetic transaminase.

^f^CrCl: creatinine clearance.

^g^ANC: absolute neutrophil count.

**Table 3:** Examples of International Classification of Diseases, Tenth Revision (ICD-10), and International Classification of Diseases, Ninth Revision (ICD-9), disease codes.

Disease	*ICD-10* codes	*ICD-9* codes
Congestive heart failure	I50.2, I50.3, I50.4	428.[2-4][0-3]
Unstable angina	I20.0	411.1
Acute myocardial infarction	I21	410.9[0-2]
Arrythmia	I49	429.9
Torsade de pointes	I45.81	426.82
Long QT syndrome	I45.81	426.82
Atrial fibrillation and flutter	I48	427.3[1-2]
Symptomatic bradycardia	R00.1	427.89
Uncontrolled hypertension	I10	401.[09]
Heart aneurysm	I25.3	414.1[09]
Coronary heart disease	I25.1	414.01
Cardiomyopathy	I42.9	425.[49­]
Vasculitis, or angiitis	I77.6	447.6
Pericardial effusion	I31.3	423.9
Peripheral vascular disease	I73.9	443.9

**Examples of normalized medical prescription (RxNORM) drug codes.**

**Drug and RxNORM code**

- Bortezomib: 356733
- Carfilzomib: 1302966
- Ixazomib: 1723735
- Lenalidomide: 342369
- Pomalidomide: 1369713

The interface uses a rule-based algorithm to match patients’ EHR data with the criteria. The comprehensive rules for matching EHR data with criteria have been described in our previous studies [37]; for instance, we defined the following rules to map each laboratory test in EHRs to 1 corresponding LOINC code:

1. Mapping the laboratory test in the LOINC dictionary to the laboratory test in the EHR, based on the popularity rank available in the LOINC dictionary
2. Mapping the laboratory test for serum or plasma samples in the LOINC dictionary to the laboratory test in the EHR when the popularity rank is not available in the LOINC dictionary
3. If one-to-one mapping is not feasible using rule 1 and rule 2, the test unit (eg, *gram* is preferred ove*molar*) is considered to facilitate the mapping
4. When one-to-one mapping is not attainable using rule 1, rule 2, and rule 3, preference is given to the laboratory test that lacks information about the method for mapping

We associated medication classes with their respective medications; for instance, we extended the annotation “post-menopausal not older than 60 years and taking LHRH [luteinizing hormone–releasing hormone] agonist” to include “post-menopausal not older than 60 years and taking goserelin, leuprolide, or other LHRH agonists.” To achieve this, we used both our in-house knowledge bases and standard resources, such as the National Comprehensive Cancer Network’s Clinical Practice Guidelines in Oncology.

Users can specify different criteria and combinations, such as different laboratory test values with specific conditions such as *no brain metastasis* to determine the number of qualified patients. The algorithm matches each patient’s EHR data with the selected criteria and calculates the number of matched patients for each criterion. The performance of the interface was evaluated by comparing it to the manual patient selection process conducted by experienced clinical domain experts.

### Ethical Considerations

This study was confirmed and approved by the Program for the Protection of Human Subjects at the Mount Sinai School of Medicine (IRB-17-01245)

## Results

### Development of Eligibility Criteria–Specific Ontology

**Figure 1:**
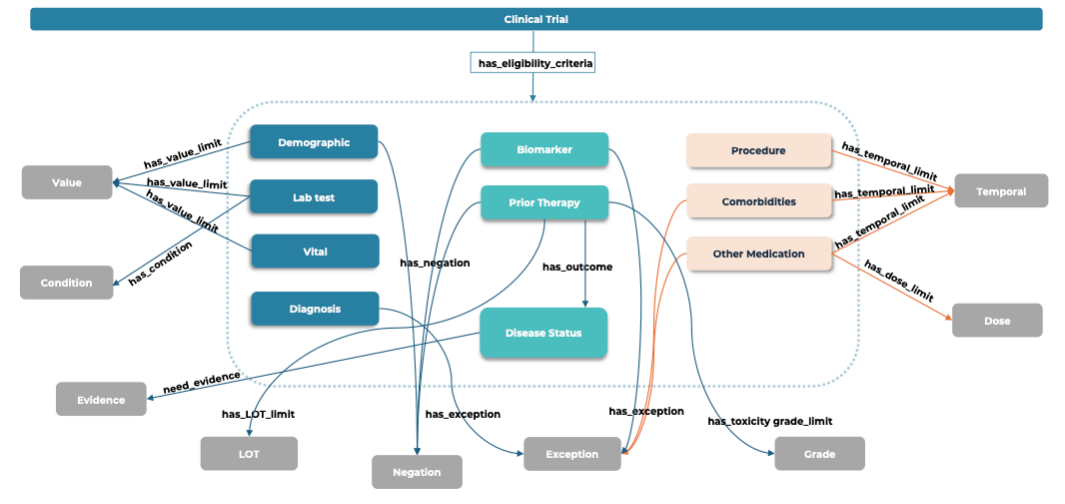
Clinical trial eligibility criteria ontology. Primary entities are grouped inside the blue dotted box. Modifier entities are placed outside the blue dotted box. The relationship between the primary entities and modifier entities always starts at a primary entity and ends at a modifier entity. LOT: line of therapy.

Our analysis of cancer clinical trials revealed that hormone therapy was the most frequently applied modality (1470/2970, 47.37%), primarily in BCa and PCa trials, followed by targeted therapy (753/2970, 25.35%) and immunotherapy (691/2970, 23.26%). Chemotherapy alone was used in 3.8% (113/2970) of the clinical trials. We developed an eligibility criteria ontology applicable to all cancer trials by manually analyzing 425 eligibility criteria (Figure 1). Entities were categorized into 10 primary groups (inside the blue dotted box) and 9 modifier groups based on semantic types and relations. Entities falling outside the blue dotted box were classified as *other observation*. The inclusion criteria mainly involved entities in the *demographic*, *diagnosis*, *laboratory test*, and *vital* groups, while the exclusion criteria commonly included entities in the *comorbidity*, *procedure*, and *other medication* groups. Entities in the *biomarker*, *prior therapy*, and *disease status* groups appeared in both the inclusion and exclusion criteria. Relationships originated from the primary groups and terminated in the modifier groups, except for the *has outcome* relationship, which started and ended in the primary group (Figure 1). To assess the applicability of the cancer eligibility criteria ontology in a different disease context, we conducted a manual analysis of 60 trials related to UC and CD. For reference, the computable formats of the manually annotated 485 trials can be found in Multimedia Appendices 4-8.

### NLP Pipeline Quality Metrics

To evaluate the quality of our NLP pipeline, we computed precision, recall, and *F*_1_-score measures. For the primary group entities, the average scores were 0.91 (precision), 0.79 (recall), and 0.83 (*F*_1_-score). Table 4 presents the range of precision, recall, and *F*_1_-score values of 17 primary group entities.

**Table 4:** Performance scores of customized natural language processing pipeline for each entity in the primary groups.

Primary group and attribute group::name			Precision		Recall		*F*_1_-score
**Demographic**							
	Demographic::age	1.000		0.923		0.960	
	Demographic::gender	1.000		0.870		0.931	
**Diagnosis**							
	Diagnosis::histology	1.000		1.000		1.000	
	Diagnosis::stage	0.667		1.000		0.800	
**Biomarker**							
	Biomarker::biomarker	1.000		0.800		0.889	
**Disease status**							
	Clinical status::disease status	0.737		0.684		0.709	
**Prior therapy**							
	Prior therapy::chemotherapy	0.944		0.895		0.919	
	Prior therapy::targeted therapy	1.000		0.786		0.880	
	Prior therapy::immunotherapy	0.897		0.788		0.839	
	Prior therapy::radiotherapy	0.682		0.682		0.682	
	Prior therapy::adjuvant therapy	1.000		0.571		0.727	
	Prior therapy::neoadjuvant therapy	1.000		0.500		0.667	
**Comorbidity**							
	Comorbidity::disease	0.842		0.762		0.800	
**Laboratory test**							
	Laboratory test::test	0.871		0.818		0.844	
**Vital**							
	Vital::vital	1.000		1.000		1.000	
**Procedure**							
	Procedure::procedure	1.000		0.600		0.750	
**Other medication**							
	Other medication::medication	0.800		0.727		0.762	

### Eligibility Criteria Attribute Extraction and Classification

The integrated named entity recognition and relation model extracted 9090 NSCLC, 7427 PCa, 10,217 BCa, 6803 MM, 1565 CD, and 1586 UC entities along with their attribute relations. After normalization and manual curation processes, the eligibility criteria knowledge base for each disease type was established in the *EntityGroup-AttributeName-Value* format (Multimedia Appendices 9-14). The number of unique *EntityGroup-AttributeName-Value* combinations varied across disease types, with 494 from 817 NSCLC trials, 471 from 649 PCa trials, 525 from 1057 BCa trials, 389 from 447 MM trials, 231 from 160 UC trials, and 230 from 151 CD trials. Notably, UC and CD trials had a smaller number of unique *EntityGroup-AttributeName-Value* combinations compared to cancer trials, indicating the presence of more complicated eligibility criteria in cancer trials.

**Figure 2:**
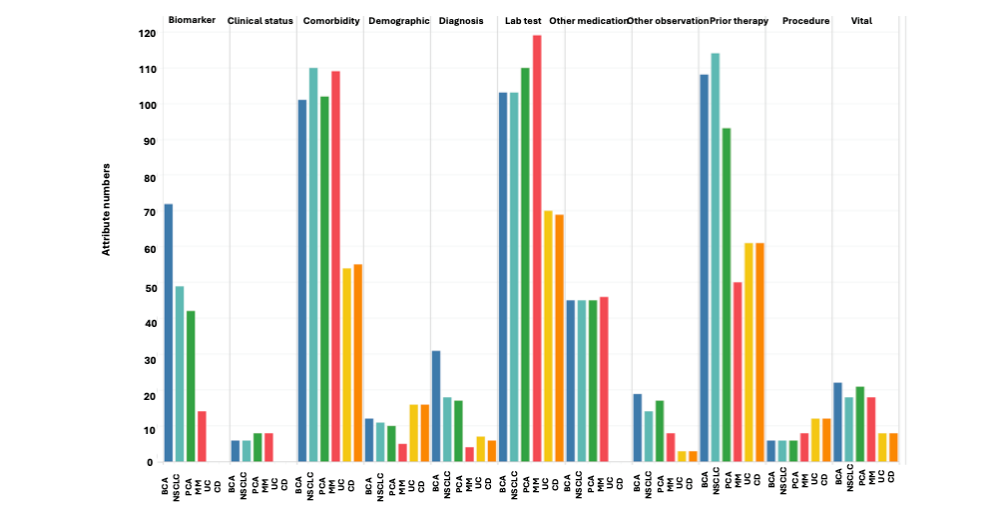
Distribution of attributes in the 10 primary groups as well as the other observation group extracted from the eligibility criteria of 4 different cancer types and 2 different autoimmune diseases. BCa: breast cancer; CD: Crohn disease; MM: multiple myeloma; NSCLC: non–small cell lung cancer; PCa: prostate cancer; UC: ulcerative colitis.

Figure 2 and Table 5 show the distribution of *EntityGroup-AttributeName-Value* combinations in each primary group from different diseases and provide examples. The *laboratory test*, *prior therapy*, and *comorbidity* groups exhibited a high number of *EntityGroup-AttributeName-Value* combinations, followed by the *biomarker* and *other medication* groups. Variations were observed between solid cancers and hematologic cancers, with higher numbers of *EntityGroup-AttributeName-Value* combinations in solid cancer types for *prior therapy* and *biomarker*, while *laboratory test* and *comorbidity* were comparable. The *diagnosis* group exhibited varying numbers of *EntityGroup-AttributeName-Value* combinations across all 4 cancer types (BCa, MM, NSCLC, and PCa). *EntityGroup-AttributeName-Value* in the *biomarker*, *diagnosis*, and *prior therapy* groups were specified per indication, while shared *EntityGroup-AttributeName-Value* were found in other primary groups.

**Table 5:** The number of attributes for 10 primary groups along with examples.

Primary group	Number of attributes					Example attributes: group, name, value (with or without condition)
	NSCLC^a^	BCa^b^	PCa^c^	MM^d^		
Demographic	11	12	10	5	Demographic, age, ≥18 y	
Diagnosis	18	31	17	4	Stage, TNM^e^ system, T2b^f^	
Biomarker	49	72	42	14	Biomarker, HER2^g^ mutation, L755P^h^	
Disease status	11	11	13	9	Disease status, relapsed, yes	
Prior therapy	114	108	93	50	LOT^i^, prior LOT, ≥2	
Comorbidity	105	96	97	108	Cardiovascular disease, arrhythmia, yes (≤3 mo)	
Laboratory test	103	103	110	119	Test, AST^j^, ≤2.5x ULN^k^	
Vital	18	22	21	18	Vital, ECOG^l^, ≥2	
Procedure	6	6	6	8	Procedure, organ transplantation, yes	
Other medication	45	45	45	46	Other medication, use of anticoagulants, warfarin (<4 wk)	

^a^NSCLC: non–small cell lung cancer.

^b^BCa: breast cancer.

^c^PCa: prostate cancer.

^d^MM: multiple myeloma.

^e^TNM: tumor, nodes, metastasis.

^f^T2b: a moderately advanced tumor in terms of size and extent but not the most advanced stage; specific implications can vary based on the type of cancer being described.

^g^HER2: human epidermal growth factor receptor 2.

^h^L755P: a reference to a specific mutation in the HER2 gene, with “L” standing for leucine, “755” being the position of the amino acid in the protein, and “P” standing for proline.

^i^LOT: line of therapy.

^j^AST: aspartate aminotransferase.

^k^ULN: upper limit of normal.

^l^ECOG: Eastern Cooperative Oncology Group.

### Transformation of Umbrella Terms Into Computable Attributes With Representative Values

#### Overview

The conversion of hypernym concepts into computable attributes along with their corresponding values was carried out. Table 6 provides some examples of converted attributes and their corresponding values for each hypernym. All lists can be found in Multimedia Appendices 9-14.

**Table 6:** Examples of hypernym concepts (entity and subgroup entity in eligibility criteria) used in eligibility criteria and converted hyponyms along with their corresponding values.

Entity and subgroup entity in eligibility criteria and converted attribute				Corresponding values
**Adequate organ function**				
	**Normal hepatic function**			
		AST^a^	≤2.5x ULN^b^	
		ALT^c^	≤2.5x ULN	
		Total bilirubin	≤1.5x ULN	
	**Normal renal function**			
		Creatinine	≤1.5x ULN	
	**Normal hematologic function**			
		ANC^d^	≥1500 cells/uL	
		Platelets	≥100,000 cells/uL	
		Hemoglobin	≥9 mg/dL	
**Comorbidities**				
	**Second malignancy**			
		All cancers	Yes, with exceptions	
	**Infectious disease**			
		HIV	Yes	
		HBV^e^	Yes	
		HCV^f^	Yes	
		TB^g^	Yes	
	**Cardiovascular disease**			
		CHF^h^	Yes	
		MI^i^	Yes	
		Angina	Yes	
		Arrhythmia	Yes	
	**Autoimmune disease**			
		UC^j^	Yes	
		CD^k^	Yes	
		Systemic lupus erythematosus	Yes	
		Rheumatoid arthritis	Yes	
		Systemic sclerosis	Yes	
		Graves disease	Yes	
		Guillain-Barré syndrome	Yes	
		Antiphospholipid syndrome	Yes	
		Sjogren syndrome	Yes	
**Biomarker**				
	**EGFR^l^ mutation sensitive to TKI^m^**			
		Exon 19 deletion	Yes	
		Exon 21 L858R	Yes	
		Exon 21 L861Q	Yes	
		Exon 18 G719C	Yes	
		Exon 18 G719X	Yes	
		Amplification	Yes	
	**EGFR mutation resistant to TKI**			
		Exon 20 T790M	Yes	
		Exon 20 C797S	Yes	
		Exon 20 S768I	Yes	
		Exon 20 insertion	Yes	
	**Mismatch repair deficient**			
		MSH2, MSH6, MLH1, PMS2, or EXO1 gene mutation	Yes	
		MLH1 hypermethylation	Yes	
**Prior therapy (targeted)**				
	**First-generation EGFR inhibitor**			
		Gefitinib	Yes	
		Erlotinib	Yes	
		Vandetanib	Yes	
	**Second-generation EGFR inhibitor**			
		Afatinib	Yes	
		Dacomitinib	Yes	
		Poziotinib	Yes	
		Tesevatinib	Yes	
	**Third-generation EGFR inhibitor**			
		Osimertinib	Yes	
		Lazertinib	Yes	
		Rociletinib	Yes	
		Tarloxotinib	Yes	
	**Proteasome inhibitor**			
		Bortezomib based	Yes	
		Carfilzomib based	Yes	
		Ixazomib based	Yes	
		Oprozomib based	Yes	
**Prior therapy (hormone)**				
	**First-generation antiandrogen**			
		Bicalutamide	Yes	
		Nilutamide	Yes	
		Flutamide	Yes	
	**Second-generation antiandrogen**			
		Abiraterone	Yes	
		Enzalutamide	Yes	
		Darolutamide	Yes	
		Apalutamide	Yes	
	**Androgen deprivation therapy**			
		Leuprolide	Yes	
		Goserelin	Yes	
		Degarelix	Yes	
	**5-α reducing agent**			
		Finasteride	Yes	
		Dutasteride	Yes	
		Megestrol acetate	Yes	
**Other medication**				
	**Current use of antibiotics**			
		Rifabutin	Yes	
		Clarithromycin	Yes	
		Azithromycin	Yes	
		Imipenem	Yes	
	**Current use of antiarrhythmic agents**			
		Propafenone	Yes	
		Procainamide	Yes	

^a^AST: aspartate aminotransferase.

^b^ULN: upper limit of normal.

^c^ALT: alanine transaminase.

^d^ANC: absolute neutrophil count.

^e^HBV: hepatitis B virus.

^f^HCV: hepatitis C virus.

^g^TB: tuberculosis.

^h^CHF: congestive heart failure.

^i^MI: myocardial infarction.

^j^UC: ulcerative colitis.

^k^CD: Crohn disease.

^l^EGFR: epidermal growth factor receptor.

^m^TKI: tyrosine kinase inhibitor.

#### Adequate Organ Function

**Figure 3:**
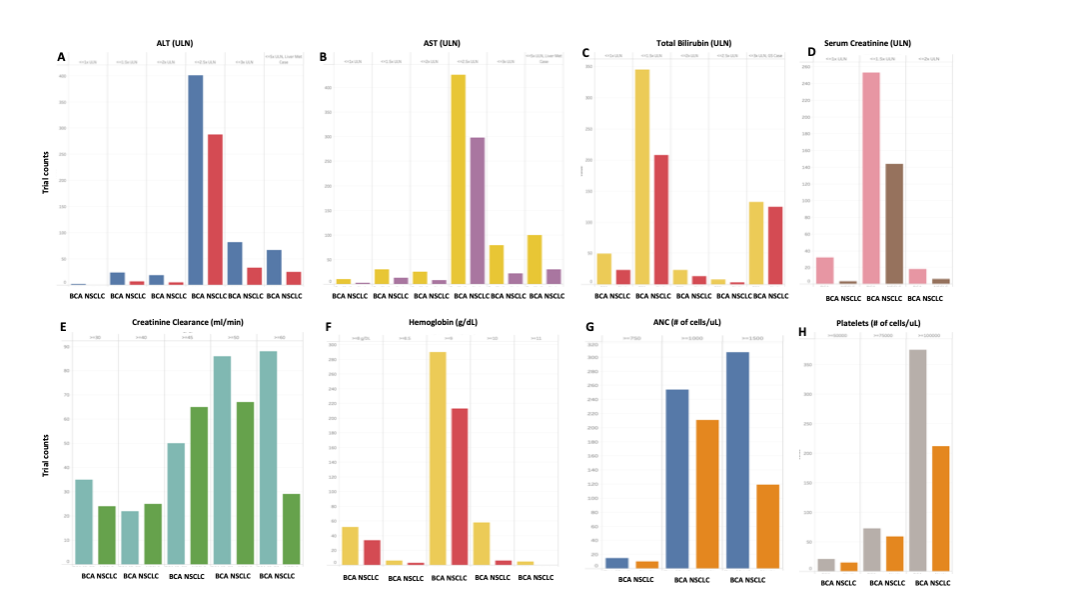
Clinical trial counts with each unique laboratory test value defining normal organ function. (A-B) Alanine transaminase (ALT) and aspartate aminotransferase (AST): normal ranges from ≤1x upper limit of normal (ULN) to ≤3x ULN, with exceptions for liver diseases (eg, liver metastasis and Gilbert syndrome [GS]) allowing values of up to ≤5x ULN. (C) Total bilirubin: normal ranges from ≤1x ULN to ≤2.5x ULN, with exceptions for liver diseases (eg, liver metastasis and GS) allowing values of up to ≤3x ULN. (D) Serum creatinine: normal ranges from ≤1x ULN to ≤2.5x ULN. (E) Creatinine clearance: normal ranges from ≥30 to ≥60 mL/min. (F) Hemoglobin: normal ranges from ≥8.0 to ≥11.0 ng/dL. (G) Absolute neutrophil count (ANC): normal ranges from ≥750 to ≥1500 cells/uL. (H) Platelets: normal ranges from ≥50,000 to ≥100,000 cells/uL. BCa: breast cancer; NSCLC: non–small cell lung cancer. For a higher-resolution version of this figure, see Multimedia Appendix 15.

Adequate organ function criteria were defined using various laboratory tests. Normal ranges and eligible values for alanine transaminase (ALT)/aspartate aminotransferase (AST), total bilirubin, serum creatinine, creatinine clearance, absolute neutrophil count, platelets, and hemoglobin were determined. Representative values for *adequate organ/hematologic function* included ≤2.5x ULN for ALT/AST, ≤1.5x ULN for total bilirubin/serum creatinine, ≥1500 cells/uL for absolute neutrophil count, ≥100,000 cells/uL for platelets, and ≥9 ng/dL for hemoglobin. Figures 3A-3H display the laboratory test value range and trial counts for each value in BCa and NSCLC clinical trials. The trends observed are similar in both cancer types.

#### Comorbidities

**Figure 4:**
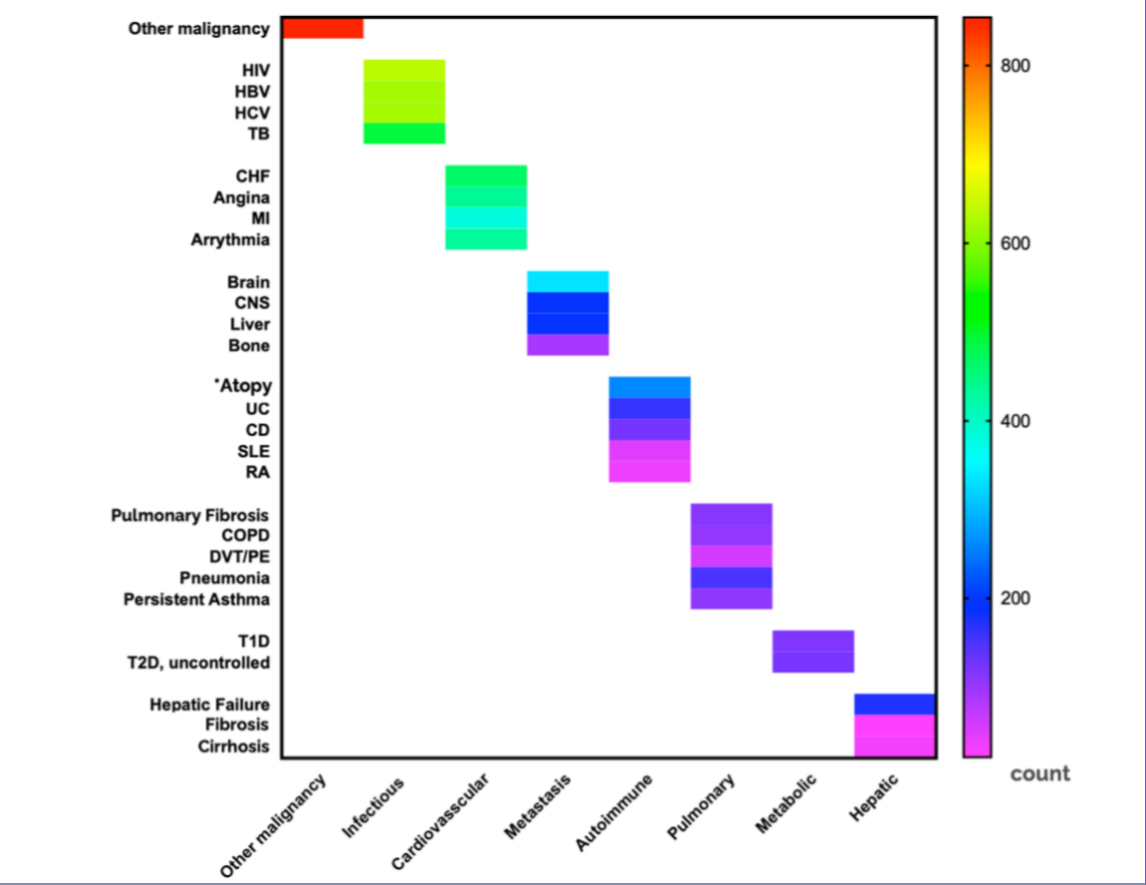
The heat map graph illustrates the number of clinical trials with each example hyponym for the hypernym comorbidities. Of note, the exception of atopy is mentioned as an autoimmune disease. The group does not include exceptions of other malignancies such as in situ cervical cancer, noninvasive bladder cancer, curative basal or squamous in situ prostate cancer, in situ breast cancer, or resected skin cancer other than melanoma. CD: Crohn disease; CHF: congestive heart failure; CNS: central nervous system; COPD: chronic obstructive pulmonary disease; DVT/PE: deep vein thrombosis/pulmonary embolism; HBV: hepatitis B virus; HCV: hepatitis C virus; MI: myocardial infarction; RA: rheumatoid arthritis; SLE: systemic lupus erythematosus; T1D: type 1 diabetes; T2D: type 2 diabetes; TB: tuberculosis; UC: ulcerative colitis.

The presence of comorbidities is a common exclusion criterion in clinical trials; however, natural language descriptions of comorbidities, such as “uncontrollable cardiovascular diseases,” “pulmonary diseases,” and “autoimmune diseases,” can be ambiguous and need domain knowledge to interpret them. We analyzed the hypernyms and their corresponding hyponyms used in BCa trial eligibility criteria. Figure 4 shows the collected hyponyms for each comorbidity class. The presence of second primary malignancies was excluded in almost all trials.

#### Prior Therapy, Other Medication, and Biomarker

By combining all examples of each hypernym, we broke down these hypernyms into actual medication and mutation hyponyms; for instance, we collected *procainamide* or *propafenone* for *current use of antiarrhythmic medication*. Similarly, we collected epidermal growth factor receptor (EGFR) exon 20 *T790M*, *T797S*, *S768I*, or *insertion* for *EGFR mutations resistant to EGFR inhibitors*.

### Development of a Prototype Interface for the Optimization of Protocol Design

**Figure 5:**
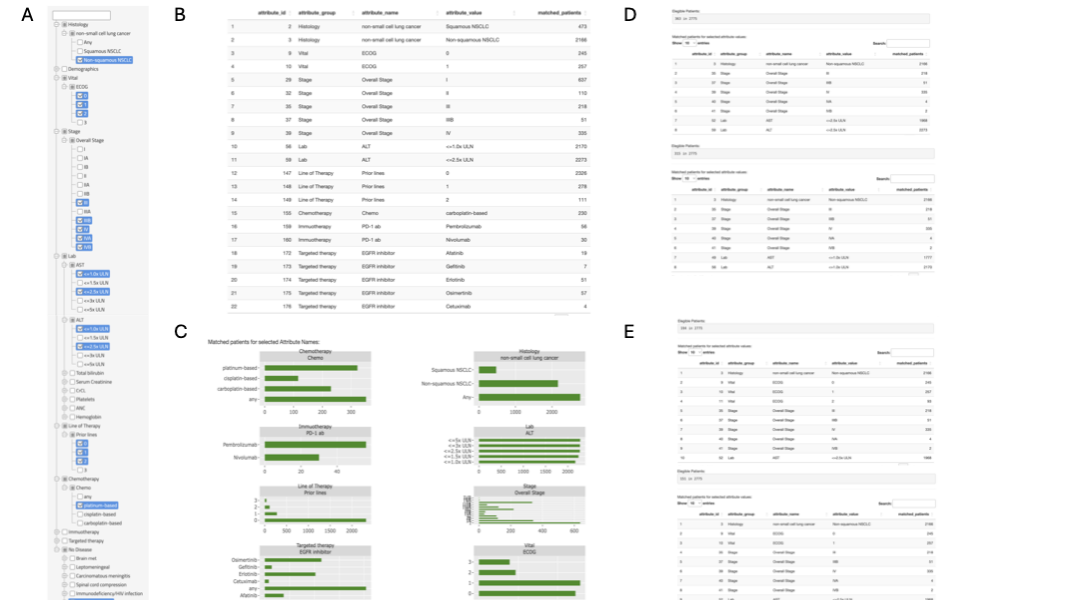
Screenshots from a prototype interface. (A-B) The selected criteria list and the corresponding number of patients. (C) The distribution of patient numbers in each group. (D) Displayed are eligible patient numbers after sequentially incorporating criteria such as non-squamous histology and stage III and IV, with the further inclusion of aspartate aminotransferase (AST) and alanine transaminase (ALT) laboratory test values of either ≤2.5x upper limit of normal (ULN) or ≤1.0x ULN. (E) The influence of Eastern Cooperative Oncology Group (ECOG) performance status as an additional criterion. Displayed are eligible patient numbers after introducing ECOG scores of 0 to 2 or 0 to 1, with histology, stage, and ALT/AST laboratory test values (<2.5x ULN) as fixed criteria. ANC: absolute neutrophil count; CrCl: creatinine clearance; EGFR: epidermal growth factor receptor; NSCLC: non–small cell lung cancer; PD-1 ab: programmed cell death protein-1. For a higher-resolution version of this figure, see Multimedia Appendix 16.

Our study investigated the impact of various criteria on the number of eligible patients. We developed a prototype interface that uses real-world patient information. Using a subset of deidentified cohorts of patients with NSCLC (n=2775), we deployed an eligibility criteria knowledge base that we had constructed in the interface. Figure 5A displays the selected criteria list, Figure 5B shows the corresponding patient number, and Figure 5C illustrates the distribution of patient numbers in each group.

Sequentially incorporating criteria such as *nonsquamous histology* and *stages III and IV* criteria, we identified 2166 (78.05%) and 426 (15.35%) eligible patients, respectively, from the total pool of 2775 patients with NSCLC. The inclusion of *AST and ALT ≤2.5x ULN* criteria yielded 363 (13.08%) eligible patients from the pool of 2775 patients. Limiting AST and ALT to ≤1.0x ULN resulted in a decreased number of eligible patients (315/2775, 11.35%; Figure 5D). In addition, we explored the influence of Eastern Cooperative Oncology Group (ECOG) performance status as an additional criterion. With histology, stage, and ALT/AST laboratory test values (<2.5x ULN) as fixed criteria, by introducing ECOG scores of 0 to 2 or 0 to 1, we identified 194 (6.99%) and 151 (5.44%) eligible patients, respectively, from the pool of 2775 patients (Figure 5E).

Patient-matching performance was evaluated using precision, recall, and *F*_1_-score performance metrics across specific clinical attributes. The average *F*_1_-score, computed across 10 attributes from 8 domains (*other primary malignancy*, *congestive heart failure*, *squamous NSCLC*, *organ/tissue transplantation*, *platelets*, programmed death*-1 antibody therapy*, *programmed cell death protein-1 or programmed cell death program-ligand 1 positive, stage groups*, *prior LOT* [line of therapy], and *ECOG*), was 0.94 (range 0.82-1.00 [37]).

## Discussion

### Principal Findings

The challenge of achieving a high success rate in clinical trials is an ongoing issue [38,39]. Our study demonstrates the feasibility of a data-driven approach to optimize trial protocols and efficiently identify eligible patients by constructing a comprehensive, EHR-interoperable eligibility criteria knowledge base and integrating EHR data. To accomplish this, we analyzed 3281 clinical trials using our customized deep learning NLP model. We extracted all entities with their attributes and converted the hypernym concepts used in eligibility criteria to EHR-compatible hyponyms along with their corresponding values. We also evaluated the feasibility of optimizing the trial protocol design on the interface we developed. This interface offers an efficient and effective approach for assessing the number of eligible patients across various combinations of eligibility criteria such as different laboratory test values as well as combinations that account for vital signs.

We developed an eligibility criteria–specific ontology by manually scrutinizing 425 eligibility criteria to be used as a reference for manual annotation during NLP model training. Accurately identifying intricate semantic relationships among entities within eligibility criteria is crucial for constructing an appropriate ontology for precise information extraction, including temporal, arithmetic values, Boolean values, and negation modifiers [31]. Our customized NLP pipeline based on the eligibility criteria–specific ontology that we created enabled us to efficiently extract all pertinent attributes across different modalities and diseases, allowing for a more accurate definition of the trial population. To determine the applicability of our ontology generated using cancer clinical trials to other disease domains, we compared the concepts and relations in clinical trials of inflammatory bowel diseases. We observed very similar trends, suggesting that our eligibility criteria–specific ontology can be extended to other types of disease trials.

Moreover, the corpus of 485 manually annotated and standardized trials in a computable format can be used in eligible patient identification in EHRs.

Liu et al [28] conducted a thorough analysis of 352,100 clinical trials across various disease domains and constructed a knowledge base of clinical trial eligibility criteria. Their comprehensive knowledge base and user-friendly interface showcased the potential of advanced NLP techniques in enhancing eligibility criteria analysis and retrieval. Fang et al [40] also adopted a data-driven approach to optimizing clinical trial eligibility criteria in the context of Alzheimer disease and pancreatic cancer domains. Building upon these efforts, our study aimed to further narrow the gap between eligibility criteria and EHRs in multicancer domains, specifically in representing the granularities of eligibility criteria for identifying eligible patients and optimizing protocol designs. This was achieved by transforming hypernyms in the criteria into EHR-compatible hyponyms. We found that most of the primary groups include umbrella terms such as *prior therapy* (eg, proper prior therapy for actionable mutations) and *biomarker* (eg, EGFR inhibitor–resistant mutations). Our study also addressed the challenge of standardizing ambiguous clinical concepts in eligibility criteria for EHR interoperability and patient matching. To overcome this challenge, we converted hypernyms to the *Entity-Attribute-Value* format using prevailing values across different cancer types and modality therapies. We believe that our EHR-interoperable standardized eligibility criteria knowledge base and interface, integrating real-world EHR data, have the potential to improve the automatic screening system. This improvement has the potential to significantly reduce manual extraction efforts. Moreover, specific, computable criteria reduce ambiguity in patient identification and enable the inclusion of a broader range of patients who may qualify for the trial but could be excluded when using more general terms. This can increase patient trial enrollment, ultimately improving the overall success rate of trials. Notably, patients who were given the option to participate in a trial by their physicians demonstrated a significantly higher participation rate of 55% [41] compared to the current average of 5% to 8% among patients with cancer [42,43]. The implementation of our *hypernym/hyponym* semantic terminology model can likewise improve the effectiveness of information retrieval from EHRs and other clinical databases in the context of real-world evidence studies.

Certain criteria such as *histology*, *stage*, *previous treatment*, or *biomarker* are difficult to modify, while others such as vital signs or laboratory test values can be adjusted during the protocol design [15]. Our study revealed the impact of modifying laboratory test values while keeping other criteria constant, resulting in fluctuations in the number of eligible patients. Our findings, which demonstrate both the number of trials for different laboratory test value ranges and eligible patient numbers, offer insights for optimizing future protocol design and refining patient selection criteria. Seeking future collaboration with clinicians to conduct a direct comparison between the patient identification results by clinical domain experts and those generated by our prototype holds promise for a more comprehensive and informative evaluation of the prototype’s performance and its potential to enhance patient identification for clinical trials. Furthermore, a careful examination of the cases identified by the prototype can provide an understanding of the nature of false positives and false negatives. This will provide insights into how the prototype may differ in its patient identification results compared to manual extraction. Our eligibility criteria knowledge base can also be leveraged for generating SCAs using EHRs. SCAs, derived from real-world evidence, are regarded as substitutes for experimental control arms in trials [16-18]. The integration of SCAs into single-arm trial data or replacing traditional control arms with SCAs can alleviate the burden of target accrual in trials with low eligible patient numbers, such as rare disease or oncology trials with specific biomarkers. The Food and Drug Administration’s approval of the palbociclib inhibitor for male patients with metastatic BCa based on real-world evidence demonstrates the potential and relevance of SCAs in improving trial design and outcomes [44].

### Limitations

Our study has several limitations to consider. First, we focused on a limited scope, analyzing only 4 different cancer types and exploring extendibility in the context of inflammatory bowel diseases. Future studies should encompass a wider range of cancer types and disease domains for a more comprehensive analysis. Second, while most attributes were well defined, some umbrella terms lacked clear examples in other cancer types, potentially affecting result accuracy. Further manual annotation using knowledge bases could enhance the precision of the attribute tables. Third, our data set may be biased because we solely included industry-sponsored trials, potentially limiting the generalizability of our findings. In addition, the NLP training and test data sets in this study can display similarities owing to the shared attributes among different cancer trials, which heightens concerns regarding potential overfitting. Fourth, we did not address entity logic, and establishing the logic between entities would enhance cohort definition accuracy. Fifth and last, our interface feasibility testing was limited to small cohorts of patients with NSCLC, and the generalizability of our findings to other populations or disease conditions may vary. Furthermore, we did not perform a quantitative evaluation of the accuracy of matched patients although domain experts checked whether the patient information matched the eligibility criteria manually. While our model serves as a valuable illustration of how NLP can contribute to the design of trials across different diseases, we fully acknowledge the indispensable role of clinicians and biomedical researchers in ensuring the integrity of trial criteria. Clinical trials vary in their objectives, encompassing assessments of treatment end points, effectiveness, and other specific goals. The process is far more nuanced than merely adjusting laboratory test values because such modifications can have a substantial impact on the pool of eligible patients. Therefore, a comprehensive approach, considering both the clinical and biomedical aspects, is imperative for robust trial design.

### Conclusions

Our study using an EHR-executable eligibility criteria knowledge base and real-world patient information provides valuable insights into the influence of different criteria on the number of eligible patients during the protocol design. The findings highlight the potential of using a data-driven approach that incorporates NLP and EHRs in clinical research.

## Appendix

Multimedia Appendix 1 Classification of attributes into primary entities and their subgroups.

Multimedia Appendix 2 Classification of attributes into modifier entities and their subgroups.

Multimedia Appendix 3 The scheme for the natural language processing–assisted clinical trial analysis.

Multimedia Appendix 4 Manually annotated non–small cell lung cancer trials.

Multimedia Appendix 5 Manually annotated breast cancer trials.

Multimedia Appendix 6 Manually annotated prostate cancer trials.

Multimedia Appendix 7 Manually annotated multiple myeloma trials.

Multimedia Appendix 8 Manually annotated ulcerative colitis and Crohn disease trials.

Multimedia Appendix 9 Non–small cell lung cancer attributes.

Multimedia Appendix 10 Breast cancer attributes.

Multimedia Appendix 11 Prostate cancer attributes.

Multimedia Appendix 12 Multiple myeloma attributes.

Multimedia Appendix 13 Ulcerative colitis attributes.

Multimedia Appendix 14 Crohn disease attributes.

Multimedia Appendix 15 Clinical trial counts with each unique laboratory test value defining normal organ function. (A-B) Alanine transaminase (ALT) and aspartate aminotransferase (AST): normal ranges from ≤1x upper limit of normal (ULN) to ≤3x ULN, with exceptions for liver diseases (eg, liver metastasis and Gilbert syndrome [GS]) allowing values of up to ≤5x ULN. (C) Total bilirubin: normal ranges from ≤1x ULN to ≤2.5x ULN, with exceptions for liver diseases (eg, liver metastasis and GS) allowing values of up to ≤3x ULN. (D) Serum creatinine: normal ranges from ≤1x ULN to ≤2.5x ULN. (E) Creatinine clearance: normal ranges from ≥30 to ≥60 mL/min. (F) Hemoglobin: normal ranges from ≥8.0 to ≥11.0 ng/dL. (G) Absolute neutrophil count (ANC): normal ranges from ≥750 to ≥1500 cells/uL. (H) Platelets: normal ranges from ≥50,000 to ≥100,000 cells/uL. BCa: breast cancer; NSCLC: non–small cell lung cancer.

Multimedia Appendix 16 Screenshots from a prototype interface. (A-B) The selected criteria list and the corresponding number of patients. (C) The distribution of patient numbers in each group. (D) Displayed are eligible patient numbers after sequentially incorporating criteria such as non-squamous histology and stage III and IV, with the further inclusion of aspartate aminotransferase (AST) and alanine transaminase (ALT) laboratory test values of either ≤2.5x upper limit of normal (ULN) or ≤1.0x ULN. (E) The influence of Eastern Cooperative Oncology Group (ECOG) performance status as an additional criterion. Displayed are eligible patient numbers after introducing ECOG scores of 0 to 2 or 0 to 1, with histology, stage, and ALT/AST laboratory test values (<2.5x ULN) as fixed criteria. ANC: absolute neutrophil count; CrCl: creatinine clearance; EGFR: epidermal growth factor receptor; NSCLC: non–small cell lung cancer; PD-1 ab: programmed cell death protein-1.

## Abbreviations

ALT: alanine transaminase
AST: aspartate aminotransferase
BCa: breast cancer
CD: Crohn disease
ECOG: Eastern Cooperative Oncology Group
EGFR: epidermal growth factor receptor
EHR: electronic health record
LOINC: Logical Observation Identifiers, Names, and Codes
MM: multiple myeloma
NLP: natural language processing
NSCLC: non–small cell lung cancer
PCa: prostate cancer
SCA: synthetic control arm
UC: ulcerative colitis
ULN: upper limit of normal

## Data Availability

The patient data sets analyzed during this study are not publicly available due to patient privacy, security and the Health Insurance Portability and Accountability Act of 1996 (HIPAA) requirement. To enable a complete run of the code shared in this study, a minimum amount of desensitized sample data could be shared with the sharing agreement. Relevant requests should be addressed to ZL. The Prototype interface is available on the internet [45]; the R scripts are available on the internet [46].
