# Supplementary figures and images for: Optimizing Clinical Trial Eligibility Design Using Natural Language Processing Models and Real-World Data: Algorithm Development and Validation
Source: JMIR AI. 2024 Jul 29;3:e50800. doi: 10.2196/50800 (PMC11319878; doi:10.2196/50800)

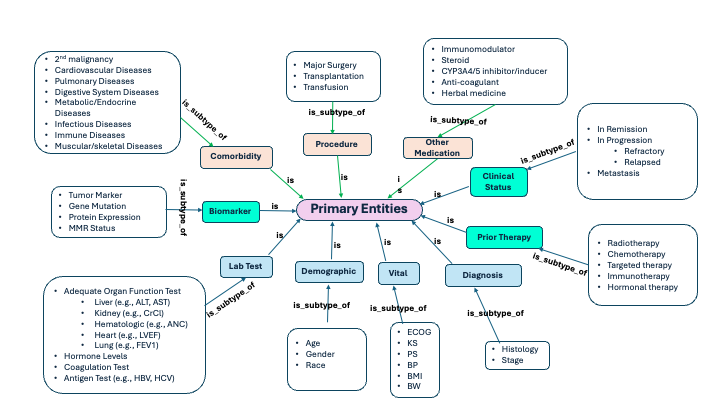

Supplement: Multimedia Appendix 1 [file ai_v3i1e50800_app1.png]

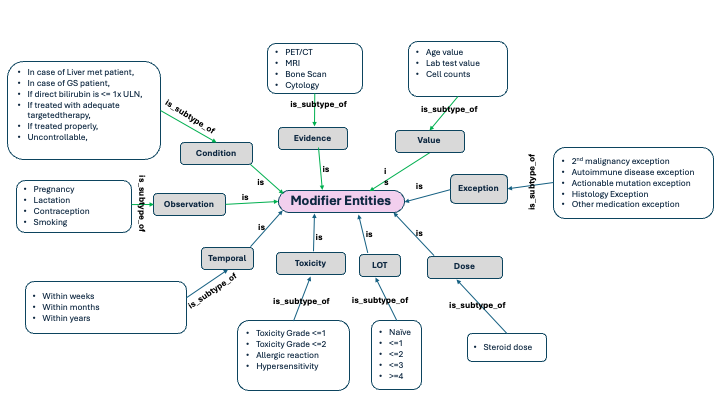

Supplement: Multimedia Appendix 2 [file ai_v3i1e50800_app2.png]

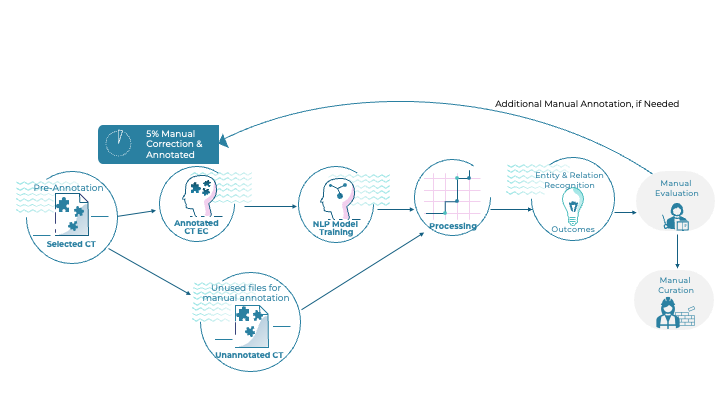

Supplement: Multimedia Appendix 3 [file ai_v3i1e50800_app3.png]

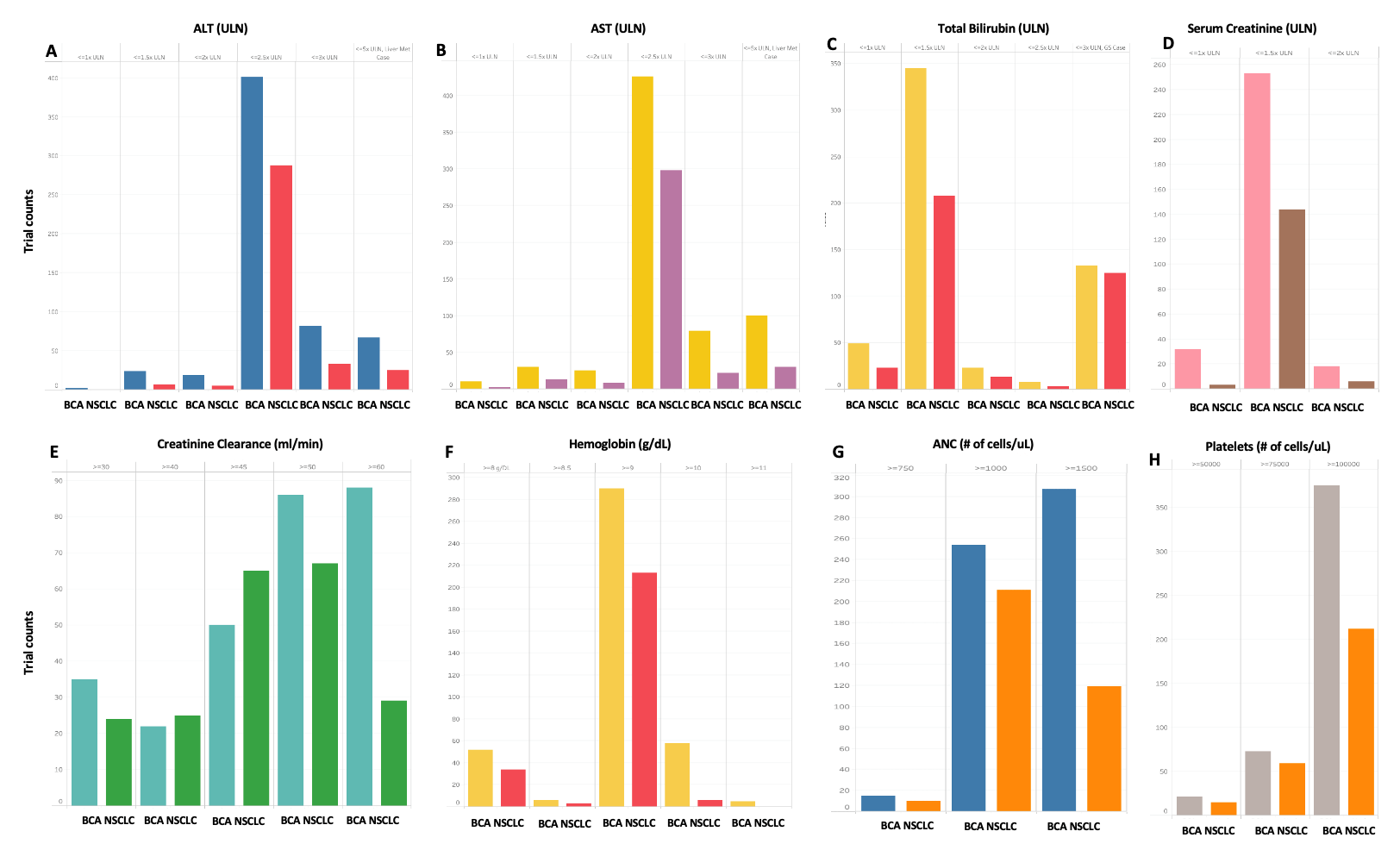

Supplement: Multimedia Appendix 15 [file ai_v3i1e50800_app15.png]

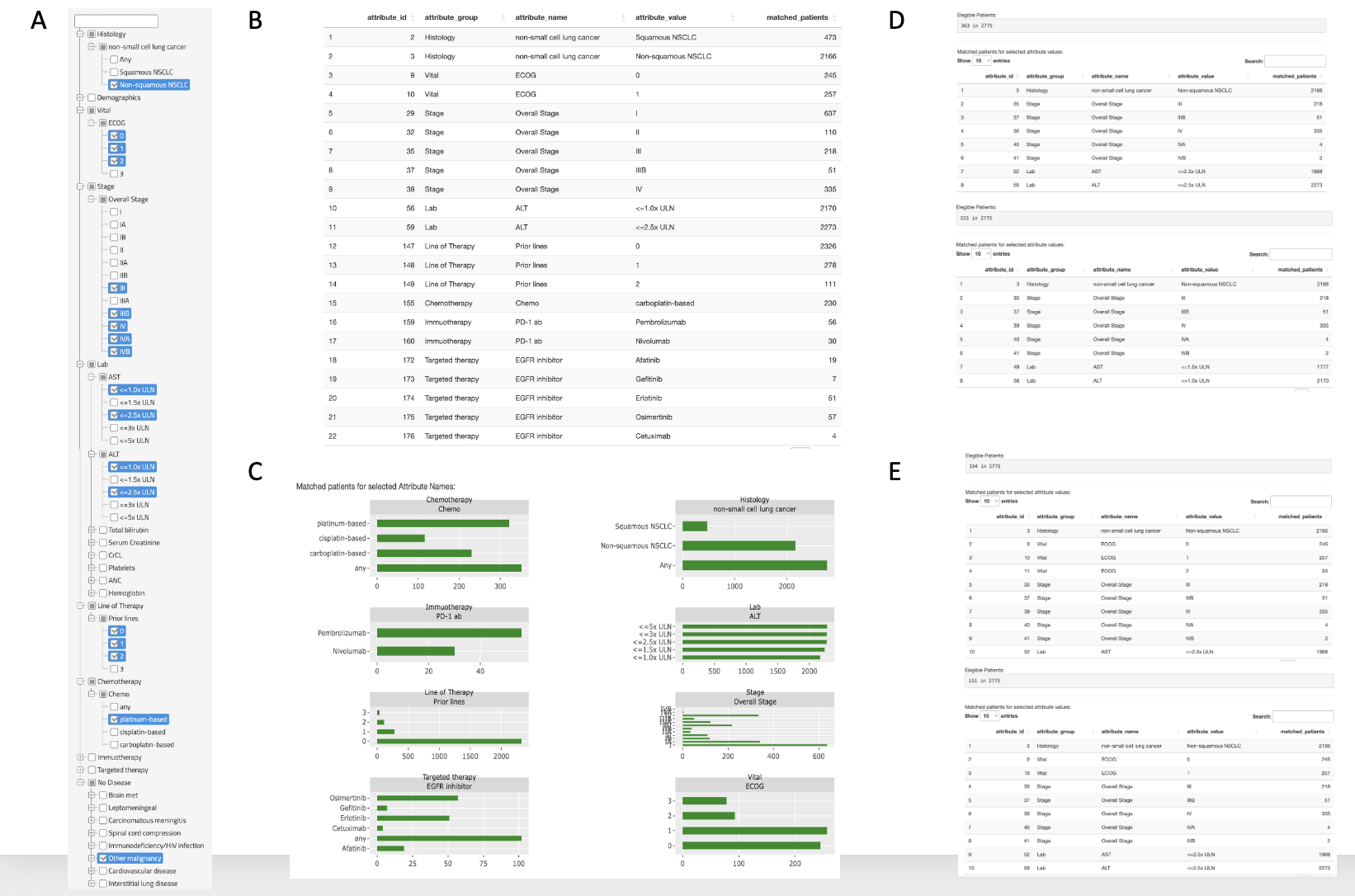

Supplement: Multimedia Appendix 16 [file ai_v3i1e50800_app16.png]
